# The migration process and temperature effect of aqueous solutions contaminated by heavy metal ions in unsaturated silty soils

**DOI:** 10.1016/j.heliyon.2024.e30458

**Published:** 2024-04-26

**Authors:** Bing Bai, Fan Bai, Jianpeng Hou

**Affiliations:** Key Laboratory of Urban Underground Engineering of Ministry of Education, Beijing Jiaotong University, Beijing 100044, PR China

**Keywords:** ADSORPTION-DESORPTION MODEL, Contaminant MIGRATION, SATURATION PROCESS, SOIL microstructure, Unsaturated SOIL

## Abstract

Adsorption-desorption experiments of three heavy metal ions (i.e., lead, copper, cadmium) in silty soil were carried out at different temperatures, and the microscopic characteristics of silty soil loaded with the three heavy metal ions were analyzed. A one-dimensional soil column was used to discuss the influences of heavy metal ion types and concentrations on the soil moisture distribution and the migration level of different heavy metal ions, especially during the dynamic change process from an unsaturated state to a saturated state. Studies show that the adsorption of heavy metal ions onto silty soil is closely related to the mineral composition and functional groups in silty soil. In addition to physical adsorption, the adsorption of heavy metal ions is closely related to the hydrolysis reaction of mineral components such as kaolinite, calcite, dolomite, plagioclase and quartz. Under constant temperature, the types and concentrations of heavy metal ions play an important role in the moisture migration of unsaturated soil. In the presence of heavy metal ions, the penetration of lead ions is the greatest, followed by copper ions and then cadmium ions. The greater the ion concentration is, the stronger the penetration of heavy metal ions in silty soils.

## Highlights

• Microscopic characteristics of a silty soil loaded with heavy metal ions were analyzed at different temperatures.
• The migration process of heavy metal ions induced by the water-saturation process was revealed.
• Three heavy metal ions in a silty soil were fitted by a typical adsorption-desorption model.
• The presence of heavy metal ions reduced the water-holding capacity of silty soils and caused the preferential flow effect.

## 1 Introduction

Heavy metal ions (HMs, such as lead, copper and cadmium) have great economic value in industrial production. However, the resulting toxicity is also extremely complex to address during production and treatment [[1], [2], [3]]. The migration and treatment of pollutants have become popular topics of current research. HM pollution in soils is usually characterized by an extended lifetime, poor detectability and accumulation in soils. At present, many scholars have studied the factors affecting the adsorption of heavy metal pollutants, including the ion concentration, temperature, and adsorption-desorption mechanism, which provide an important reference for follow-up geotechnical environmental treatment [[4], [5], [6]]. In addition, many scholars have conducted in-depth research on the establishment of nonlinear adsorption-desorption models such as the typical Freundlich model and Langmuir model [7] and the analysis of the adsorption-desorption mechanism from microscopic characteristics [8,9].

Chen et al. [10] showed that the adsorption behavior of silt to lead ions is mainly attributed to the ion exchange between lead ions and feldspar, while the adsorption behavior of loess is mainly the interface precipitation between calcite and lead ions, and the maximum adsorption capacity of loess is much higher than that of silty soils. Jurate et al. [11] showed that there were obvious differences in the migration processes of different heavy metals through adsorption tests of Cr^2+^, Pb^2+^, Cu^2+^, and Zn^2+^ on soils. Niu et al. [12] indicated that the adsorption behavior of copper ions by geomaterials is essentially an internal thermal process of entropy increase. The adsorption performance of copper ions on nanoporous silica materials is better, and its adsorption performance is linearly related to the pH value of the suspension. The tests of Sun et al. [13] on saturated sand columns showed that the adsorption performance of kaolinite for Pb^2+^ is significantly higher than that of sandy soils; therefore, the addition of kaolinite can clean the lead ions on the surface of sandy soils, and as a carrier, it can promote the migration of lead ions. Gray et al. [14] showed that the pH value, organic matter and cation exchange capacity have obvious effects on the adsorption and desorption behavior of Cd^2+^. According to the investigation of a farm in Spain, Covelo et al. [15] found that the adsorption priority of clay minerals in soils to heavy metals is Pb^2+^>Cu^2+^>Cd^2+^>Ni^2+^>Zn^2+^. Peld et al. [16] showed that pH has a significant effect on the desorption behavior of apatite NO^3−^ and Cl^−^ but a weak effect on the deposition behavior.

The mechanism of the migration of soluble pollutants is via the redistribution of moisture in the pores as a carrier in soils. Seepage flow in porous media plays a leading role and is accompanied by the migration and adsorption of heavy metal pollutants [[17], [18], [19]]. In fact, the transport mechanism of HMs includes multiple effects, such as convection, molecular dispersion and mechanical dispersion [[20], [21], [22]]. Whether in unsaturated or saturated soils, the molecular diffusion coefficient of pollutants is closely related to the volumetric water content [[23], [24], [25]]. If the seepage flow in the pores is quick, the effect of mechanical dispersion is greater than that of molecular dispersion, and the effect of molecular diffusion can be neglected. Conversely, if the seepage velocity of pore water is small, only the effect of molecular dispersion can be considered. In addition, the migration of HMs is also affected by the physical characteristics of soil particles, pollutant characteristics and external environmental factors, which deserve attention [[26], [27], [28]].

In this paper, static adsorption-desorption tests of HMs on silty soil as a porous medium were carried out, and the adsorption mechanism of typical HMs was discussed from the physical properties and microstructure characteristics of functional groups, chemical elements and mineral compositions. The effects of temperature, types of HMs and ion concentration on the adsorption-desorption characteristics were explored, and a nonlinear adsorption-desorption model was verified. Finally, through a one-dimensional unsaturated soil column permeability test, the saturation process of water and the migration mechanism of HMs were analyzed when water was injected with HMs. In addition, the effects of the concentration and HM types on the moisture content and the distribution characteristics of HMs in the soil column were given, and the physical essence of the proposed adsorption-desorption model was revealed considering the adsorption lag effect.

## 2 Materials and methods

### 2.1 Test soil

**Fig. 1:**
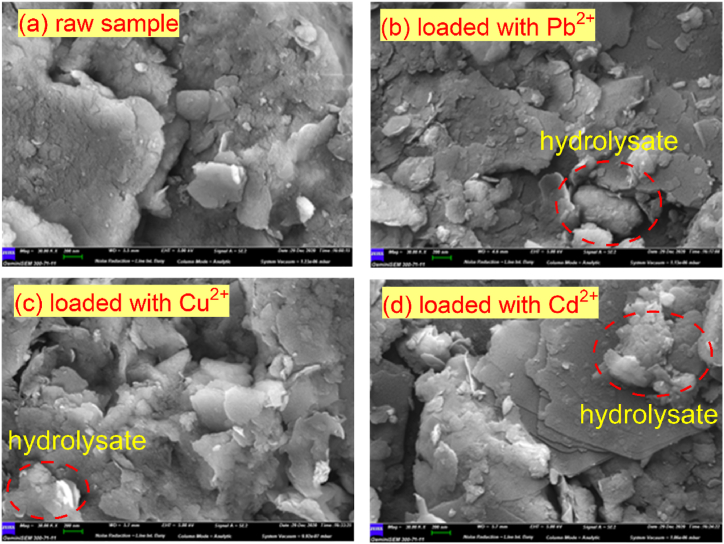
Microstructures of silty soil: (a) raw sample, (b) loaded with Pb^2+^, (c) loaded with Cu^2+^, and (d) loaded with Cd^2+^.

The silty soil used in the test was taken from Lingshou County, Hebei Province, China. The limit moisture contents, such as the liquid limit, plastic limit and plasticity index of silt, were 20.9 %, 12.2 %, and 8.7, respectively. The specific gravity of the particles was 2.71, and the maximum dry density and optimum moisture content were 1.51 g/cm^3^ and 0.16 %, respectively. A laser scattering particle size analyzer (LA-950, dry method) produced by HORIBA was used to measure the particle size distribution, and its range was 0.1–100 μm, with an average particle size of 30.5 μm. The microstructure images of raw silt obtained by a Hitachi 4800 scanning electron microscope (SEM) are shown in Fig. 1 (a), and its specific surface area is 28.32 m^2^/g. The mineral compositions of silt determined by using a SmartLab-9KW XRD system are quartz (24 %), illite (18 %), kaolinite (6 %), calcite (2 %), plagioclase (15 %), microcline (14 %), hematite (2 %), and montmorillonite (19 %). Through the EDS energy spectrum test (Hitachi 4800), the main constituent elements of silt were obtained: O (48.1 %), C (39.7 %), Si (5.0 %), Al (3.8 %), Fe (1.4 %), K (0.2 %), Ca (0.2 %) and Cl (0.1 %).

### 2.2 Heavy metal ions

Lead, copper and cadmium ions were selected as typical heavy metal pollutants. The lead ion solution (i.e., Pb (NO_3_)_2_) is a colorless transparent liquid obtained by dissolving lead powders with 5 % nitric acid. Copper ion solution was obtained by dissolving blue rhombohedral crystals of anhydrous copper nitrate Cu(NO_3_)_2_·6H_2_O (Beijing Inokai Technology Co., Ltd.) in deionized water. A cadmium ion solution was obtained by dissolving the white prismatic crystal cadmium nitrate tetrahydrate Cd(NO_3_)_2_·4H_2_O (Beijing Inokai Technology Co., Ltd.) in deionized water.

A TAS-900 graphite furnace atomic spectrophotometer was used to determine the concentrations of lead and cadmium ions in a solution. By measuring the absorbance of Pb^2+^ and Cd^2+^ after the adsorption reaction was completed and according to their calibration curves, the concentrations of Pb^2+^ and Cd^2+^ were obtained by conversion [29,30]. The concentration of copper ions in a solution cannot be determined by atomic spectrophotometry. However, it can be indirectly obtained by using an ultraviolet spectrophotometer (T45 N, produced by Jinghua Technology Instrument Co., Ltd.) to measure the complex resulting from the reaction between the solution and copper reagent (DDTC-Na), which is thin white film crystals. In addition, the complex is a yellow‒brown colloid and can remain stable for 30 min, which facilitates the determination.

### 2.3 Test apparatus

**Fig. 2:**
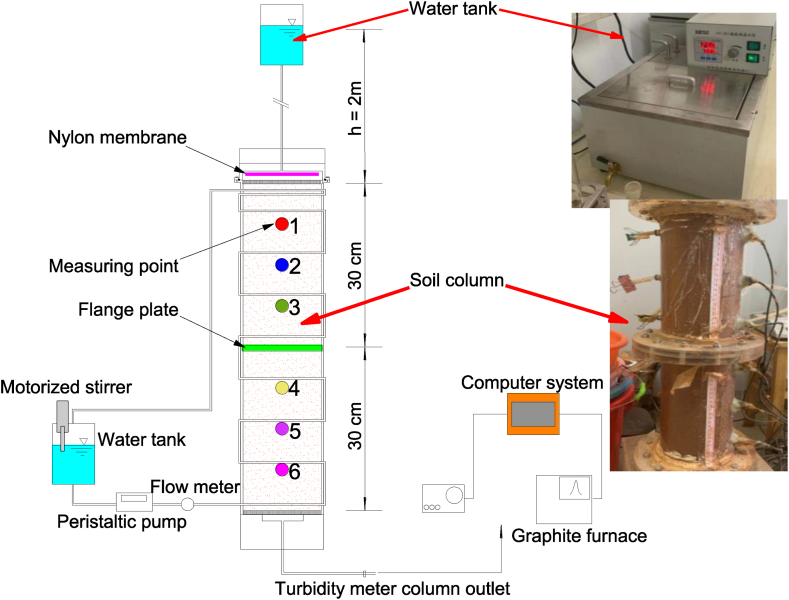
Experimental apparatus and photos.

The one-dimensional unsaturated soil column device (Fig. 2) consists of a test cylinder, thermostatic control device, data acquisition system and water supply system. The length, inner diameter and wall thickness of the test cylinder are 60, 14.8 and 0.6 cm, respectively. Moreover, the test cylinder is composed of two high temperature resistant acrylic cylinders with a length of 30 cm connected by flanges and bolts, and both ends are also connected with covers by flanges and bolts to provide a constant water head to the soil column. In addition, two rows of holes with the same height and a vertical interval of 10 cm are reserved in the sidewall of the cylinder to easily arrange the lead-out of the moisture sensors, temperature sensors in the soil column and samples of contaminated soil. Both the thermostatic control system and the water supply system consist of a water tank and heat-resistant rubber hoses. Regarding the thermostatic control system, a constant temperature was set through the water tank (HH-600, temperature control range: 20 °C−100 °C, produced by Lichen Technology Company), and a peristaltic pump was used to introduce the internal water of the tank into the hoses wrapped around the outer wall of the test cylinder to form a circulating water flow to achieve a constant ambient temperature [31]. Regarding the water supply system, the water tank was used to provide seepage water with a constant temperature. The data acquisition system includes sensors and collectors that were used measure the temperature and humidity. The PT100 platinum resistance temperature sensor has the characteristics of high sensitivity, high stability and strong oxidation resistance. Its probe diameter is 2 mm with a length of 15 mm, and the temperature measurement range is −70 °C–500 °C with an accuracy of ±0.5 °C. The temperature collector (JY-DAM-TC16) is a thermocouple acquisition module. An EC-5 soil moisture sensor with a measurement accuracy of 0.1 % and a ZL6 soil water collector produced by American Meter Group Company were used as the moisture sensor and water collector, respectively.

Furthermore, the test results of the moisture sensors are greatly affected by temperature changes. To calibrate the measured value of volume moisture content, a specific small-scale sealed soil column sample (ensuring the same moisture content and dry density) was used for calibration; that is, the test readings could be calibrated by adjusting the temperature of the soil sample (temperature range: *T* = 20−60 °C) and measuring the readings of the moisture sensor at the same time. The test showed that there is a linear fitting relationship between the readings of the moisture sensor and the temperature. That is, if the temperature at State 1 is *T*_1_ and the moisture sensor reads *θ*_1_ and the temperature at State 2 is *T*_2_ and the sensor reads *θ*_2_, the actual volume moisture content change during this period shall be $d\theta =\left(\theta_{2}-\theta_{1}\right)-0.0012\times \left(T_{2}-T_{1}\right)$. Regarding the adsorption test of HMs to silty soil, the solution was prepared and oscillated with a double-layer vibrator (HY-6A, produced by Jintan Zhengrong test instrument company) for 1 h (110 times/min) to make HMs fully react with particles and form a stable adsorption state before measurement.

### 2.4 Adsorption-desorption test method

The silt was placed in an oven at a set temperature of 105 °C for 12 h until it was completely dry. Several 0.2-g soil samples were weighed with disposable weighing paper and prepared with different concentrations of lead, copper and cadmium ion solutions in dry and clean conical bottles. Then, these samples were placed in a vibrator for 1 h (110 times/min). According to the adsorption kinetics process test that was performed in advance, the adsorption time was set to 24 h, and after the adsorption-desorption reaction, the pollutant solution concentration of each group of samples was measured three times. The average value was then taken as the final value to ensure the test reliability. Regarding the adsorption process, the concentration range of the solution was set to 0−1200 μg/ml, while the concentration range of the solution in the desorption process was set to 80−800 μg/ml, and three different temperatures were selected (i.e., 20, 40 and 60 °C). In addition, regarding the desorption process at a certain concentration (e.g., 800 μg/ml), the adsorbed HMs were desorbed gradually by adding deionized water (i.e., by decreasing the concentration or by implementing a dilution process) until the solution concentration approached 0.

To determine the differences in the microstructure of silt loaded with HMs, two concentrations of 50 μg/ml and 100 μg/ml for Pb^2+^, Cu^2+^ and Cd^2+^ solutions were specially prepared and mixed into beakers containing 5 g of silt for 24 h. Then, the silt samples were filtered out by a vacuum filter pump for the measurement of SEM photos after drying.

### 2.5 Migration test of HMs in unsaturated soils

The initial volume moisture content of the soil column was set to 15.8 %, and the dry density was 1.3 g/cm^3^. To ensure a uniform distribution of moisture in the soil column, silt filling was carried out in 12 layers along the height of the test cylinder (5 cm per layer). After each layer of soil was filled and compacted to the target height, the surface layer was shaved using a scrape and then filled with the next layer of soil. After the soil column was completed, covers at both ends were connected with the flanges on the test cylinder by bolts. The measuring points of the moisture/temperature sensor were marked as 1, 2, 3, 4, 5 and 6 from top to bottom (Fig. 2), and the holes were sealed with glass glue after the arrangement was completed to ensure that no water leakage occurred during the seepage process.

The initial temperature of the soil column was 20 °C. If necessary, the soil column temperature could be increased through the thermostatic control device to reach the target temperature (e.g., *T* = 40, 60 °C). According to the injected solution concentration, seepage migration tests were carried out in five cases: deionized water, Pb^2+^ solution (20, 100 μg/ml), Cu^2+^ solution (100 μg/ml) and Cd^2+^ solution (100 μg/ml). The tests were conducted with the soil column placed vertically and a constant head of *h* = 2 m to ensure one-dimensional seepage conditions. Of course, the influence of gravity cannot be ignored in the discussion of the mechanism.

As the seepage tests began, the temperature, moisture content and concentration of HMs in the filtrate were measured at the same time, and after completion, samples were taken at 7 different positions of the soil column (Measuring Points 1, 2, 3, 4, 5 and 6 and the infiltration location). For the same sampling point, 3 samples were determined, and the average value was taken as the final value.

The concentrations of HMs in the soil samples were determined as follows: 100 ml of EDTA solution with a concentration of 0.15 mol/L was mixed into a conical flask containing 3 g of dried soil sample, and the oscillator was then used for 24 h to make it fully chelate with Pb^2+^, Cu^2+^, and Cd^2+^ adsorbed in the soil to extract the HMs. The extracted solution was put into a centrifuge tube and run for 5 min until the supernatant was transparent. Then, its adsorption capacity was measured.

## 3 Results and discussion

### 3.1 Static adsorption-desorption process and theoretical description

#### 3.1.1 Lead ions

**Fig. 3:**
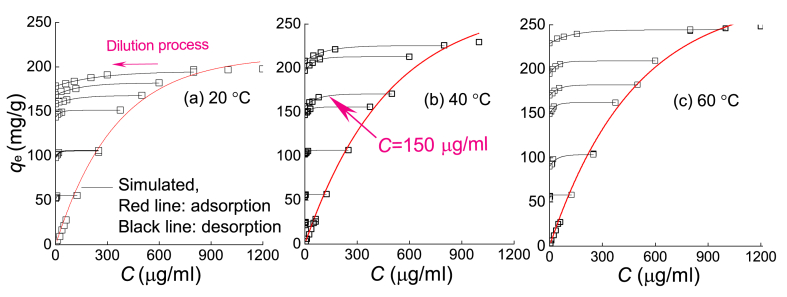
Adsorption-desorption curves of Pb^2+^: (a) 20 °C, (b) 40 °C, and (c) 60 °C.

According to the SEM image of the silt (Fig. 1 (a)), there are many bulges and depressions on the surface of the soil particles, which have a large specific surface area, making the silt easy to combine with the HMs in the solution and thus showing high adsorption efficiency. In fact, the experiments carried out by the authors showed that the adsorption rate of Pb^2+^ was faster at low solution concentrations, and the adsorption efficiency was more than 90 %. With an increasing Pb^2+^ concentration, the adsorption rate decreases, and the adsorption capacity gradually reaches the maximum value. For example, the adsorption efficiency of Pb^2+^ decreased to 32.97 % at a concentration of 1200 μg/ml from 90.56 % at a concentration of 12.511 μg/ml. Fig. 3 shows that the characteristics of the Pb^2+^ adsorption-desorption curves were basically the same at different temperatures (20, 40 and 60 °C; see Fig. 3(a), (b) and (c), respectively), and the adsorption rate started to decrease as the solution concentration increased and the adsorption amount tended to be constant. The desorption rate at the initial stage was slow and tended to accelerate (e.g., 150 μg/ml in Fig. 3(b)) until the Pb^2+^ concentration decreased to a certain value. This is because when the desorption reaction takes place in silt, the concentration of residual Pb^2+^ in the solution is still greater than that on the solid particles, although there is more Pb^2+^ at the high concentration adsorption point. At this time, the solubility product equilibrium constant is far from being reached. Therefore, the Pb^2+^ adsorbed in the silt is difficult to desorb, and only the physically adsorbed Pb^2+^ is released. As the Pb^2+^ concentration in the solution continues to decrease, the adsorption amount on the solid particles is gradually greater than the Pb^2+^ concentration, resulting in an increase in the adsorbed Pb^2+^ released from the silt and a faster desorption efficiency.

The adsorption amount was calculated using the batch equilibrium method (Eq. (1)).

(1) $$q_{e}=\frac{\left(C_{0}-C_{e}\right)\cdot V}{m}$$

where *q*_e_ is the adsorption capacity of soil to pollutants, *C*_e_ is the residual concentration of the solution after a reaction occurs, *C*_0_ is the initial concentration, *V* is the volume of solution, and *m* is the mass of soil.

The model considering the adsorption history was used for fitting the adsorption-desorption process (Fig. 3), and the relationship between the concentration *C* and the adsorption capacity *q*_e_ can be expressed by Eq. (2) (i.e., Bai model) [31].

(2) $$\frac{\partial q_{e}}{\partial C}=\left\{ \begin{array}{c} k_{d}\cdot {\;e}^{{-\beta }_{1}\cdot \frac{C}{C_{L}}\;}，\;\frac{\partial C}{\partial t}>0\\ k_{r}\cdot {\;e}^{-\beta_{2}\cdot \frac{C}{C_{L}}}，\;\frac{\partial C}{\partial t}<0 \end{array}\right.$$

where *k*_d_ and *k*_r_ are the equilibrium constants in the adsorption and desorption processes, respectively [M^−1^L^3^], *β*_1_ and *β*_2_ are the corresponding attenuation factors [−], *C* is the injection concentration, and *C*_L_ is a characteristic value.

The static adsorption-desorption parameters obtained by fitting are shown in Table S1, indicating that the proposed model could describe the behavior of Pb^2+^ on silt well, with a determination coefficient of *R*^2^ > 0.969. The adsorption-desorption curves at different temperatures in Fig. 3 (e.g., the initial Pb^2+^ concentration is 800 μg/ml) were also compared. This comparison showed that the adsorption amount of Pb^2+^ gradually increased with an increasing temperature, that is, 193.98, 225.560, and 242.86 mg/g for temperatures of 20, 40, and 60 °C, respectively, and the adsorption efficiency also increased at the same concentration. At this time, the corresponding adsorption equilibrium constant *k*_d_ increased (i.e., 0.5694, 0.5746 and 0.5925 at 20, 40, and 60 °C, respectively), while the desorption equilibrium constant *k*_r_ gradually decreased to 0.4735, 0.4601 and 0.4338, respectively.

This can be attributed to the increase in temperature improving the internal energy of Pb^2+^ so that its activation energy changes to the transition state [32], which makes Pb^2+^ easier to adsorb by silt. This result is consistent with the endothermic reaction in the adsorption process of Pb^2+^ given by Niu et al. [12]. When the desorption reaction occurred (Fig. 3), the increase in temperature also improved the residual adsorption capacity of Pb^2+^; that is, the adsorption capacity of silt for Pb^2+^ was improved. Actually, the increase in temperature strengthens the water turbulence in soils, and the thermodynamic force also increases due to the stochastic Brownian motion of HMs. The higher the temperature is, the higher the collision probability between soil particles and HMs will be. As a result, the more sufficient the interaction between the two substances is, the stronger the adsorption effect.

#### 3.1.2 Copper ions

Fig. S1 shows that the maximum adsorption capacity of Cu^2+^ was significantly lower than that of Pb^2+^, which was observably different from the adsorption-desorption of Cu^2+^ by quartz sand given by Bai et al. [31]. The increase in the concentration of Cu^2+^ also increased the adsorption capacity of Cu^2+^ to silt. Moreover, the test results showed that when the concentration of Cu^2+^ was 10–60 μg/ml, its adsorption capacity was 4.792–39.184 mg/g with an adsorption efficiency of more than 90 %. While the concentration of Cu^2+^ continued to increase, the adsorption efficiency began to decrease, which is similar to the case observed for Pb^2+^.

As shown in Table S1, the attenuation factors in the adsorption process and desorption process were *β*_1_ = 0.0066 (*R*^2^ = 0.995) and *β*_2_ = 0.0940 (*R*^2^ = 0.945), respectively. The corresponding attenuation coefficients of Pb^2+^ were *β*_1_ = 0.0027 (*R*^2^ = 0.998) and *β*_2_ = 0.0714 (*R*^2^ = 0.945), indicating that the nonlinear characteristic of Cu^2+^ was more obvious than that of Pb^2+^. In the previous investigation by Bai et al. [31], the adsorption between Cu^2+^ and quartz sand was mainly caused by the hydrolysis reaction of Cu^2+^, and the product of copper hydroxide precipitation was attached to the surface of quartz sand through dispersion. Moreover, the desorption of Cu^2+^ is caused by water flow cleaning, which indicates obvious linear characteristics. Silt is composed of multiple mineral components and has a larger specific surface area. Therefore, the Cu^2+^ adsorbed on the silt surface is significantly greater than that of quartz sand. Moreover, in addition to physical adsorption, obvious chemical adsorption occurred. For this reason, the Cu^2+^ attached to the silt is difficult to peel off in the desorption process, resulting in obvious nonlinear characteristics.

Similar to Pb^2+^, the amount of adsorbed Cu^2+^ during the adsorption process improved with an increasing temperature, with the same trend of the adsorption equilibrium remaining constant. However, the desorption equilibrium constant was the opposite. This is because the adsorption of Cu^2+^ is an endothermic reaction and the desorption reaction is an exothermic reaction; hence, the increase in temperature will inhibit the desorption reaction of Cu^2+^ [12,32].

#### 3.1.3 Cadmium ions

The adsorption-desorption characteristics of Cd^2+^ were similar to those of Pb^2+^ and Cu^2+^. Similarly, the increase in temperature gradually increased the adsorption amount of Cd^2+^ (Fig. S2), and the desorption equilibrium constant of Cd^2+^ changed from 0.1055 to 0.0737 after the temperature rose from 20 °C to 60 °C (Table S1), which is related to the endothermic reaction of the adsorption behavior of Cd^2+^. However, the influence of temperature variation on the desorption reaction is significantly lower than that on the adsorption reaction, so its residual adsorption capacity is also significantly higher than that at room temperature.

The proposed nonlinear adsorption-desorption model improved the traditional isothermal adsorption-desorption models and can reflect the effects of temperature and heavy metal types and the adsorption-desorption history described by the equilibrium constants and attenuation coefficients well.

### 3.2 Adsorption-desorption mechanism

According to the comparison of Fig. 3 and Figs. S1 and S2, the adsorption amount of Pb^2+^ was the largest, followed by that of Cu^2+^ and then that of Cd^2+^. For the solution concentration of 800 μg/ml at 20 °C, the maximum adsorption capacities were 193.976, 88.854 and 75.672 mg/g, respectively. During the desorption process, the maximum residual adsorption amount also decreased gradually to 178.363, 70.358 and 56.9571 mg/g, respectively.

Fig. 1(b), (c) and (d) show that when silt adsorbed HMs, the microstructures of the soil particle surface changed significantly. Specifically, the effect of Pb^2+^ caused the flaky structure (red circle: hydrolysate) on the surface of soil particles, the effect of Cd^2+^ caused the superposition of the flaky structure, while the effect of Cu^2+^ caused the flocculent phenomenon to be more serious, resulting in the reduction of the specific surface area of soil particles (compared with the SEM image of the raw sample; Fig. 1(a)). Relevant research has shown that the adsorption of Pb^2+^ and Cd^2+^ is mainly an ion reaction, while the adsorption of Cu^2+^ is mainly ion exchanges [30]. Therefore, the silt loaded with Cu^2+^ is obviously different from that of Pb^2+^ and Cd^2+^.

#### 3.2.1 Lead ions

**Fig. 4:**
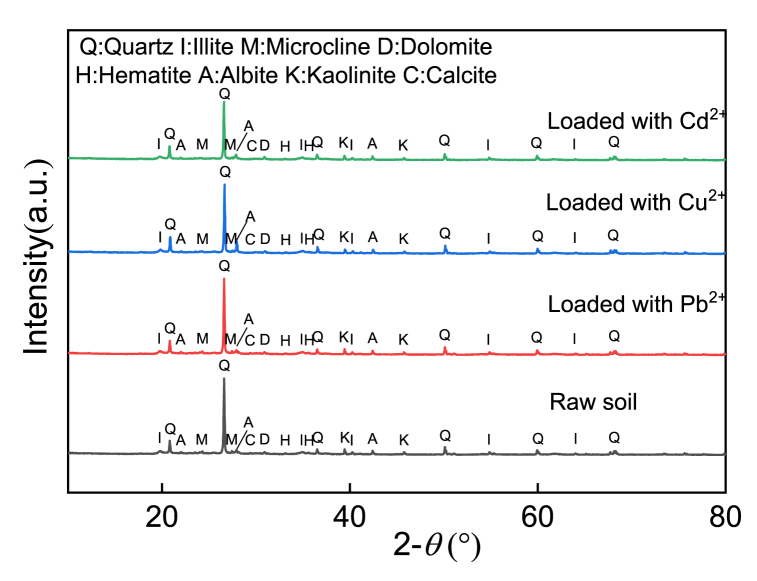
XRD infraction pattern of the silty soil loaded with (a) Pb^2+^, (b) Cu^2+^, and (c) Cd^2+^.

**Fig. 5:**
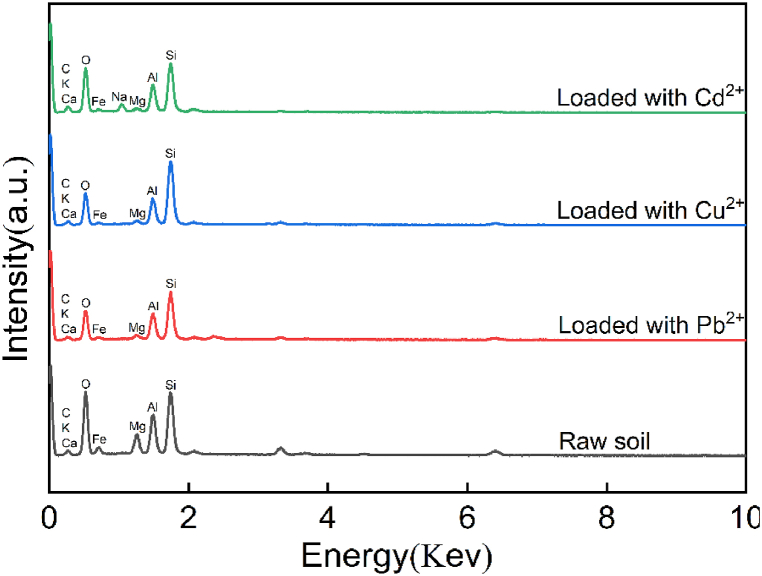
EDS spectrum of the silty soil loaded with (a) Pb^2+^, (b) Cu^2+^, and (c) Cd^2+^.

A Pb^2+^ solution with pH = 7 was used for the adsorption test, and it was found that the pH of the solution after the test was less than 7. In the XRD diffraction pattern (Fig. 4, red curve), the diffraction peaks of calcite in silt decreased after the adsorption of Pb^2+^. In the EDS spectrum (Fig. 5, red curve), the content of O significantly decreased, and the contents of Ca and Mg also decreased, indicating that Pb^2+^ reacted with part of the calcite [[33], [34], [35]]. The reaction process can be represented by Eqs. (3), (4), (5).

(3) $$H_{2}O+Pb^{2+}\to \text{Pb}{\left(\text{OH}\right)}^{+}+H^{+}$$

(4) $$\text{Pb}{\left(\text{OH}\right)}^{+}+\text{CaC}O_{3}\to \text{PbC}O_{3}+Ca^{2+}+OH^{-}$$

(5) $$\text{Pb}{\left(\text{OH}\right)}^{+}+\text{CaMg}{\left(CO_{3}\right)}_{2}\to 2\text{PbC}O_{3}+Ca^{2+}+2OH^{-}+{\text{Mg}}^{2+}$$

Compound analysis by XRD mapping revealed that a small amount of Pb_2_SO_4_(OH)_2_ existed in the soil sample. In addition, the contents of Mg, Fe, O, Al and Si were relatively reduced according to EDS energy spectrum analysis. In fact, clay minerals contain anisotropic interlayer charges, which can lead to coordination reactions, as expressed in Eqs. (6), (7) or cation exchange (Eq. (5)). The XRD diffraction pattern also revealed that the composition of kaolinite, plagioclase and other minerals decreased; hence, HMs were held by clay minerals in the form of complexes.

(6) $$2\equiv \text{SOK}+Pb^{2+}\to \equiv \text{SPbOS}+2K^{2+}$$

(7) $$2\equiv SO^{-}+Pb^{2+}\to \equiv \text{SOPbOS}$$

where *S* represents the mineral composition and *K* represents the HMs held by clay minerals.

**Fig. 6:**
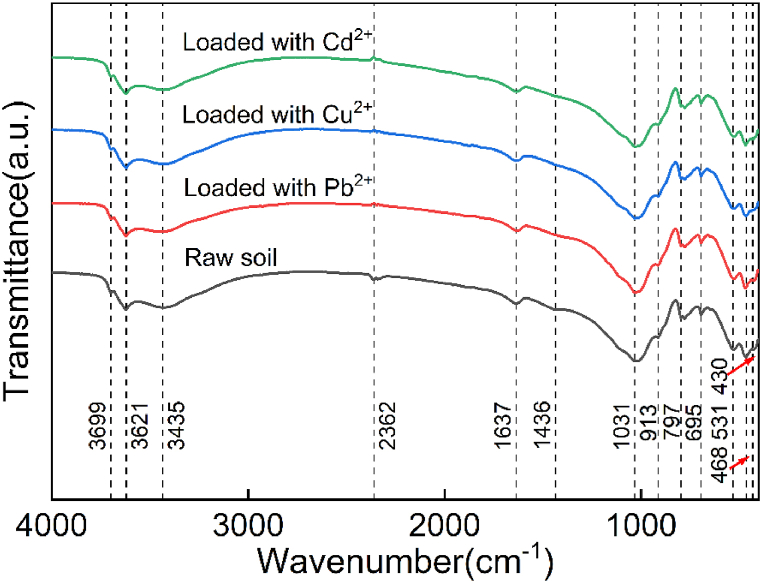
FT-IR functional group chart loaded with (a) Pb^2+^, (b) Cu^2+^, and (c) Cd^2+^.

In the FT-IR spectrum (Fig. 6, red curve), the wave peak at 1436 cm^−1^ disappeared after the silt was loaded with Pb^2+^, indicating that the –COOH contained in kaolinite was combined with Pb^2+^. In addition, the wave peak at 430 cm^−1^ disappeared, which was related to the decrease in the relative content of Si after loading with Pb^2+^. Fig. 4 (red curve) shows that the corresponding peaks of 68.82° and 59.94° in XRD decreased after the adsorption of Pb^2+^. Although the Si–O bond in quartz was relatively stable [[16], [36]], its crystal surface might also undergo a hydrolysis reaction (Eq. (8)) to form a precipitate.

(8) $$\equiv \text{Si}-O-\text{Si}\equiv +\text{Pb}{\left(\text{OH}\right)}^{+}+H_{2}O\to \equiv \text{Si}-O-\text{Pb}\left(\text{OH}\right)+\equiv \text{Si}\left(\text{OH}\right)+H^{+}$$

#### 3.2.2 Copper ions

Fig. 4 (blue curve) shows the XRD diffraction pattern of silt after loading with Cu^2+^, which indicates that the diffraction peak characteristics at 20.88°, 27.94° and 59.96° corresponding to illite, albite and kaolinite, respectively, were obviously stronger. Moreover, new diffraction peaks appeared at 12.32° and 41.14°, demonstrating that the mineral composition in the silt played a key role in the adsorption of Cu^2+^. The diffraction peak value of calcite decreased at 65.32°, which showed that Cu^2+^ reacted with part of the calcite [37]. The pH value of the solution after adsorption was 6.85, which is less than 8. According to the solubility product balance, the equilibrium constant for generating precipitation was not reached, so Cu(OH)_2_ cannot be generated. The peak value of calcite in the XRD diffraction pattern and the relative content of O decreased. This phenomenon exhibits by Eq. (9).

(9) $$\text{CaC}O_{3}+Cu^{2+}\to \text{CuC}O_{3}+Ca^{2+}$$

Fig. 5 (blue curve) shows that the content of Mg decreased; that is, the adsorption reaction on illite and kaolinite generated new complexes. The adsorption mechanism of copper ions can be expressed by Eqs. (10), (11).

(10) $$\equiv \text{SOH}+Cu^{2+}⇌\equiv \text{SO}{\text{Cu}}^{2+}+H^{+}$$

(11) $$\equiv \text{SOH}+Cu^{2+}+H_{2}O⇌\equiv \text{SOCuOH}+2H^{+}$$

When Cu^2+^ was not adsorbed, the silt had weak absorption peaks at 2362 cm^−1^ (Fig. 6, blue curve). After Cu^2+^ was adsorbed, these two absorption peaks disappeared. Moreover, the absorption peak at 1436 cm^−1^ in the raw sample disappeared after the adsorption of Cu^2+^, which was related to the carboxyl functional groups. The interaction relationship between copper ions and carboxyl groups can be expressed by Eq. (12).

(12) $$-\text{COOH}+Cu^{2+}\to -\text{COOC}u^{+}+H^{+}$$

#### 3.2.3 Cadmium ions

For Cd^2+^, the transmittance at 3699 and 3621 cm^−1^ represented the presence of –OH functional groups in the clay minerals of the silt (Fig. 6, green curve), 1031, 797, and 695 cm^−1^ represented Si–O functional groups, and 531 and 468 cm^−1^ represented Si (Al) functional groups. These kinds of functional groups did not change after adsorption of Cd^2+^, indicating that these functional groups did not react with Cd^2+^. This is also consistent with the fact that there is no obvious downward trend in the content of Si in Fig. 5 (green curve). The transmissivity at 3435 and 1637 cm^−1^ was caused by the stretching vibration of clay mineral molecules after the adsorption of water. After loading with Cd^2+^, the wavenumber did not significantly change, indicating that the functional groups herein were not related to the reaction with Cd^2+^. The reason for these peaks may be related to the bound water in silt as well as the adsorbed water from air. In particular, it should be noted that the 1436 cm^−1^ absorption peak in the silt disappeared after the adsorption of Cd^2+^, which was related to the bending vibration of the carboxyl functional group –COOH in the mineral (Eq. (13)).

(13) $$-\text{COOH}+Cd^{2+}\to -\text{COOC}d^{+}+H^{+}$$

Compared with the raw sample, the diffraction peaks of silt at 24.24°, 29.4°, 24.22°, 26.729°, 27.72°, 28.22°, 29.4° and 50.1° decreased after the adsorption of Cd^2+^ (Fig. 4, green curve). Obviously, the strengthening of the diffraction peak at 27.88° was related to albite, kaolinite and illite. Kaolinite has a point of zero charge of 5 and will adsorb Cd^2+^ at pH > 4, while the pH after the adsorption of Cd^2+^ is 6.8. Additionally, it can be seen in Fig. 5 (green curve) that the elements Si, Al and O have decreased to a certain extent. Therefore, Cd^2+^ reacts with silicate acid minerals to form complexes of CdSiO_3_ and CdAl(SiO_4_)_2_. The reaction process can be represented by Eqs. (14), (15).

(14) $$S\equiv \text{OH}⇌=SO^{-}+H^{+}$$

(15) $$S\equiv O^{-}+{\text{Cd}}^{2+}⇌=\text{CdS}O^{-}$$

where *S* refers to the clay minerals in silt.

The potentials of silt under the action of Pb^2+^, Cu^2+^ and Cd^2+^ were 1.76, −9.64, and −11.3 mv, respectively. For raw silt, its zeta potential energy was −7.78 mv. For most clay mineral particles, the surface of soil particles was negatively charged due to their own mineral compositions. Because of ion reactions, coordination reactions and complexation reactions, the silt surface was loaded with obvious HMs. However, different HMs react differently with silt, and the final potential is also different. In general, the order of potential is Pb^2+^, Cu^2+^ and Cd^2+^, which is consistent with the order of adsorption capacities in the static adsorption-desorption test presented in Section 3.

The microscopic changes demonstrated that the adsorption-desorption process is closely related to the types of chemical reactions (e.g., ion reaction, ion exchange) and the changes in the electric potential and functional groups.

### 3.3 Migration of the aqueous solution contaminated by HMs

#### 3.3.1 Influence of HMs on the movement of moisture

**Fig. 7:**
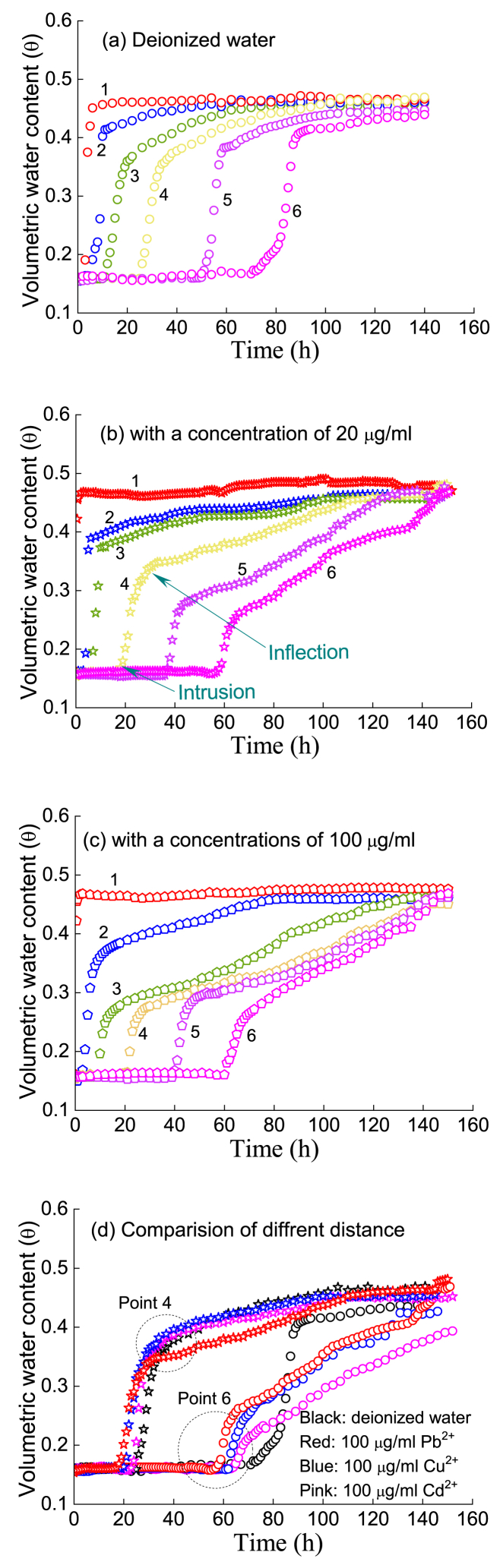
Movement process of moisture with time in the presence of the Pb^2+^ solution: (a) deionized water; (b) 20 μg/ml, (c) 100 μg/ml, and (d) comparison of different distances.

**Fig. 8:**
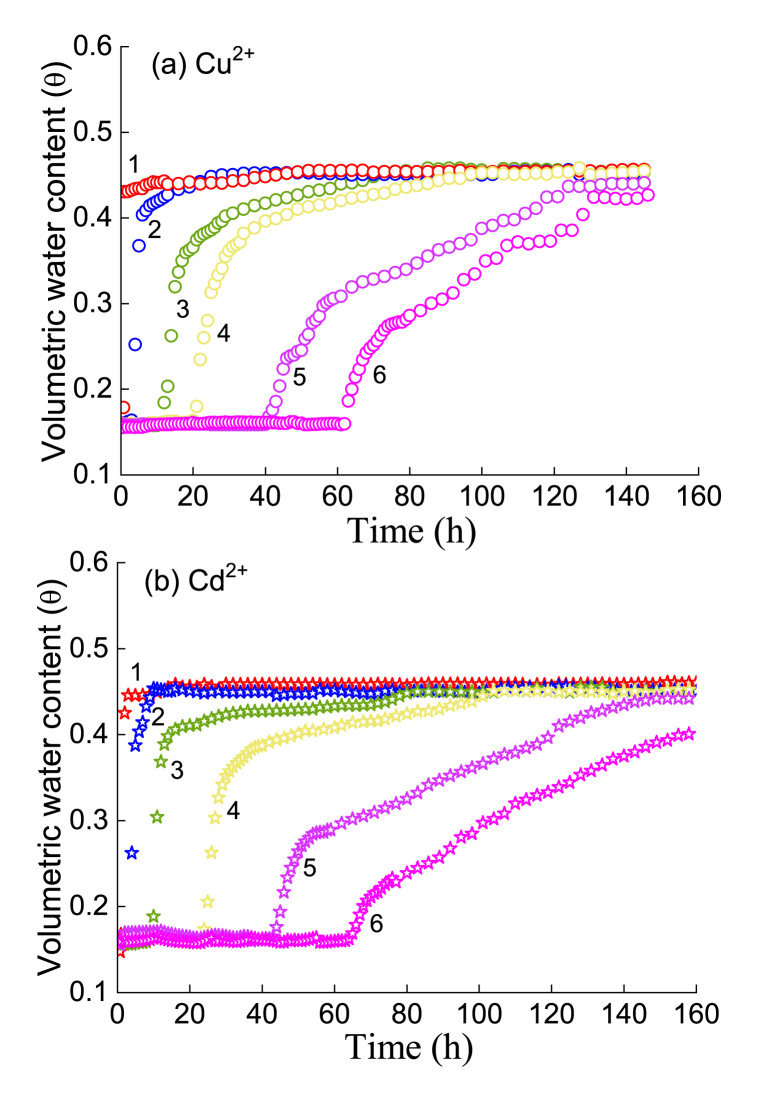
Movement process of moisture with time with a concentration of a 100-μg/ml solution: (a) Cu^2+^ and (b) Cd^2+^.

Fig. 7, Fig. 8 show the variation in moisture with time when deionized water, Pb^2+^, Cu^2+^ and Cd^2+^ solutions were injected into an unsaturated silty soil column, and the moisture content of the soil column will continue to increase and transform, especially from an unsaturated state to a saturated state. For the case of injecting deionized water (Fig. 7(a)), the peak volume moisture content gradually reached a further distance from the infiltration surface (i.e., measuring points 1, 2, 3, 4, 5 and 6 in Fig. 2) after 140 h. For the case of injecting Pb^2+^ solution (Fig. 7(b), (c)), Cu^2+^ solution (Fig. 8(a)) and Cd^2+^ solution (Fig. 8(b)), there were similar evolution rules.

For the same HM (e.g., Pb^2+^), the greater the concentration of the injected solution is, the faster the infiltration front reaches the same measuring point. For example, regarding Point 4, when the concentration gradually increased (Fig. 7; e.g., 0 → 20→100 μg/ml), the time at which the intrusion point reached Point 4 was 25, 19 and 17 h, and the time at which it reached the inflection point was 38, 36 and 28 h. Moreover, the further the measuring point was from the injection surface, the faster the wetting front reached the point compared with the deionized water flow. Furthermore, the earlier the inflection point of moisture content appears, the lower the inflection point value of moisture content.

In fact, the surface of soil particles loaded with HMs will change; that is, the solute potential of HMs on water is enhanced, and HMs react with mineral components, which weakens the water holding capacity of soil particles. Thus, during the process of water absorption, the soil more easily flows from the position with low matrix suction to the position with high matrix suction. In other words, preferential flow will be generated during the migration of HMs, and the infiltration front will reach the measuring point in a shorter time.

The comparison test of the same concentration and different types of HMs (Fig. 7, Fig. 8) showed that the penetration of Pb^2+^ was the highest, followed by Cu^2+^, and Cd^2+^ was the lowest, where Points 4 and 6 are considered characteristic points. This result is consistent with the previous static adsorption test results in Section 3.

#### 3.3.2 Migration process of HMs

**Fig. 9:**
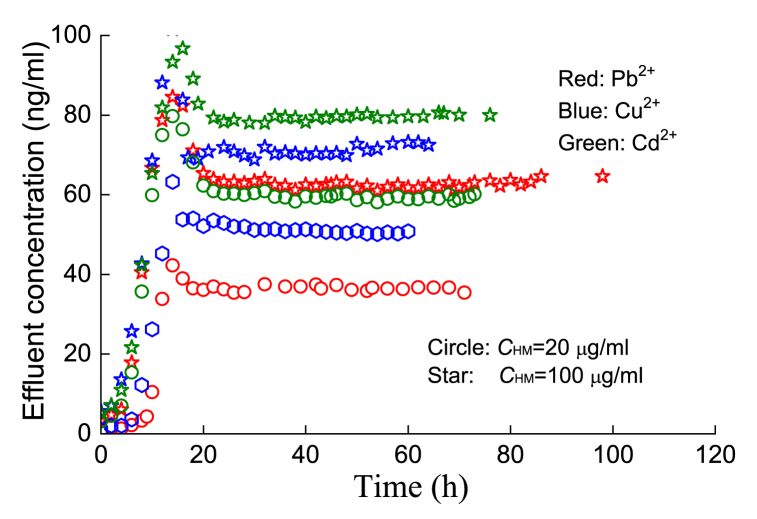
Migration processes of the three HMs at different injected concentrations.

In Fig. 9, when the injection concentration of HMs was 100 μg/ml, the effluent concentration was less than 100 ng/ml; that is, the adsorption of HMs by silt was basically more than 90 %. Obviously, the greater the amount of adsorbed HMs in soil there are (e.g., Pb^2+^ compared with Cd^2+^), the smaller the penetration time and the peak concentration of the filtrate. Under the same injection HM concentration (e.g., *C*_HM_ = 100 μg/ml), the greater the adsorption capacity of HMs, the more obvious the influence will be on the surface characteristics of soil particles, which in turn causes the water holding capacity of soil particles to be weaker and the suction capacity of the matrix to decrease, resulting in preferential flow and an earlier filtration time. In fact, the concentrations of heavy metal ions selected for the experiments were much higher than those found in commonly contaminated soils, so silty soil has potential as a long-term adsorbent.

When the wetting front reached the bottom of the soil column and the soil changed from unsaturated to saturated [38,39], the HMs mainly passed through the path with small matrix suction formed by the presence of HMs [40]. Therefore, the HM concentration first tended to increase at the initial stage, e.g., in the time range of 0–15 h, and reached an effluent peak. However, when the soil was gradually saturated, the HMs percolated along the internal path of the porous medium. At this time, the outflow concentration decreased rapidly and then reached a steady state. This is a key turning point that affects the migration characteristics of HMs. The experimental results indicated that the presence of HMs alters the interface characteristics between soil particles (Fig. 4, Fig. 5, Fig. 6) and ultimately affects the migration process of the filtrate. Of course, the presence of HMs also significantly changes the matrix suction of unsaturated soil, which is an important factor in water migration. Certainly, the effect of temperature cannot be ignored (Fig. 3; Figs. S1 and S2). The quantitative measurement of matrix suction of soils loaded with different HMs at diff erent temperatures is crucial for the establishment of migration theory models and is a topic worthy of attention in the future.

In addition, the effluent concentration of HMs improved with an increasing injection concentration (20 → 100 μg/ml), and the peak concentration of the breakthrough curve was also high. In fact, according to the previous static adsorption-desorption test, when the injection concentration is 100 μg/ml, the unit mass of soil is far from reaching the adsorption saturation point.

#### 3.3.3 Temperature influence on migration processes

**Fig. 10:**
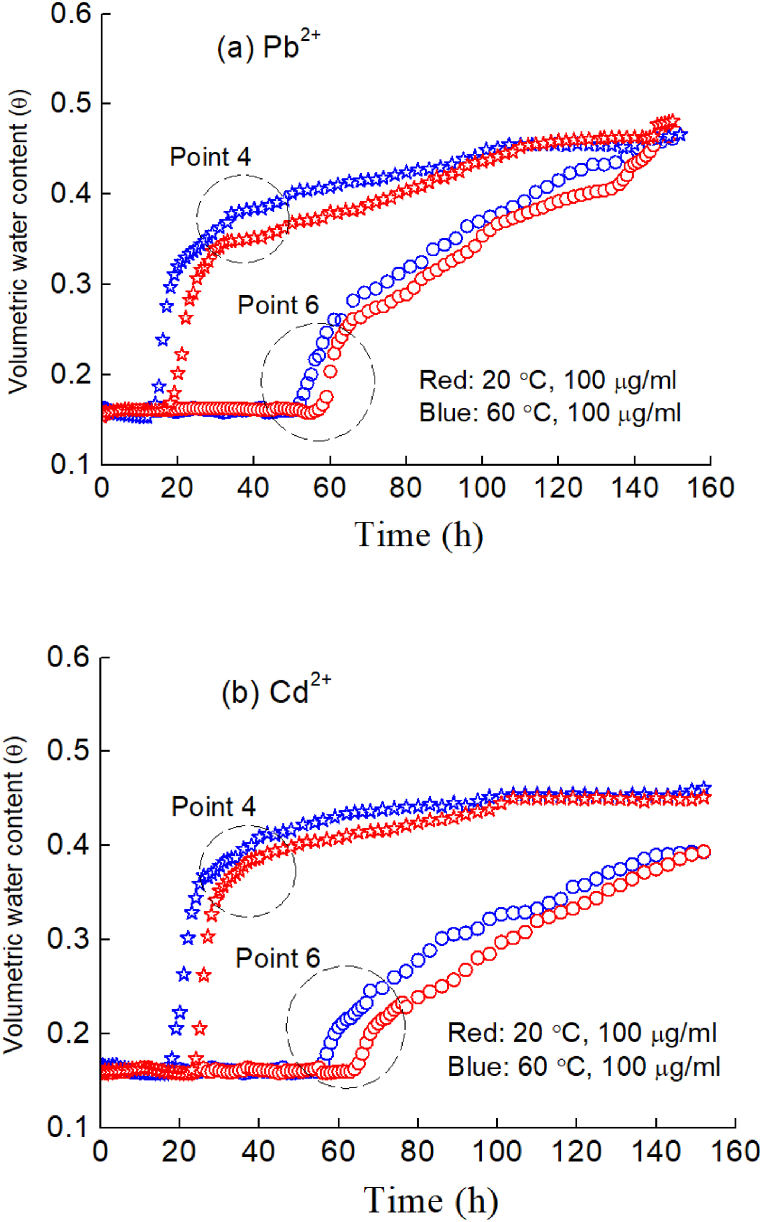
Temperature influence on the moisture migration processes at different temperatures: (a) loaded with Pb^2+^and (b) loaded with Cd^2+^.

For clarity, Fig. 10 only shows the moisture movement patterns of two characteristic points (points 4 and 6) in the soil column when there existed HMs in the injected water (loaded with Pb^2+^ and Cd^2+^; see Fig. 10(a) and (b), respectively). From Fig. 10, as the temperature increases (i.e., 20 °C–60 °C), the internal energy of water molecules accelerates, and simultaneously, the water in the soil is easily converted from bound water to free water, indicating a clear thermodynamic driving force. Therefore, the migration of moisture is significantly accelerated, meaning that at the same time, the moisture content in the soil decreases significantly.

**Fig. 11:**
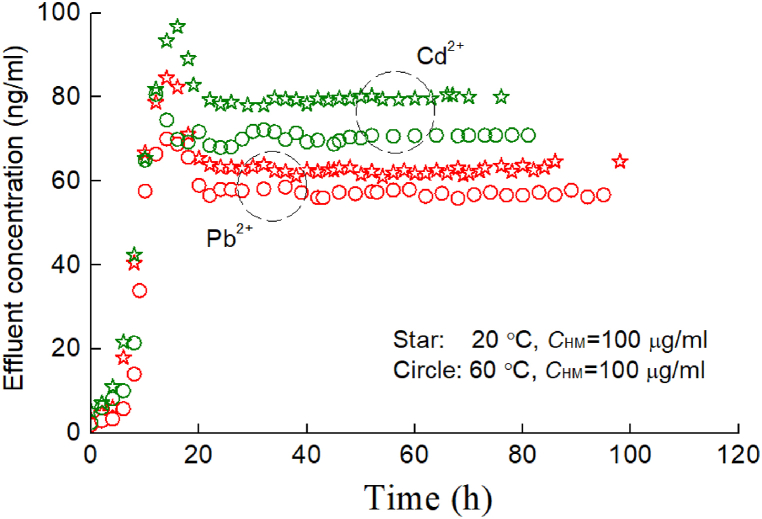
Temperature influence on the HM migration at different temperatures.

However, the increase in temperature enhances the adsorption performance of heavy metal ions and soil particles (see Fig. 1, Fig. 3), resulting in a decreasing trend in the concentration of heavy metal ions (e.g., Pb^2+^ and Cd^2+^; Fig. 11) in the effluent. From Fig. 11, at an injected concentration of a 100 μg/ml solution, this gap between 20 °C and 60 °C is approximately between 10 % and 15 %, and the temperature effect cannot be ignored. Obviously, due to the greater adsorption performance of Pb^2+^ with soil particles than Cd^2+^, the concentration of Pb^2+^ in the effluent of the soil column is lower.

#### 3.3.4 Distribution of HMs along the soil column

**Fig. 12:**
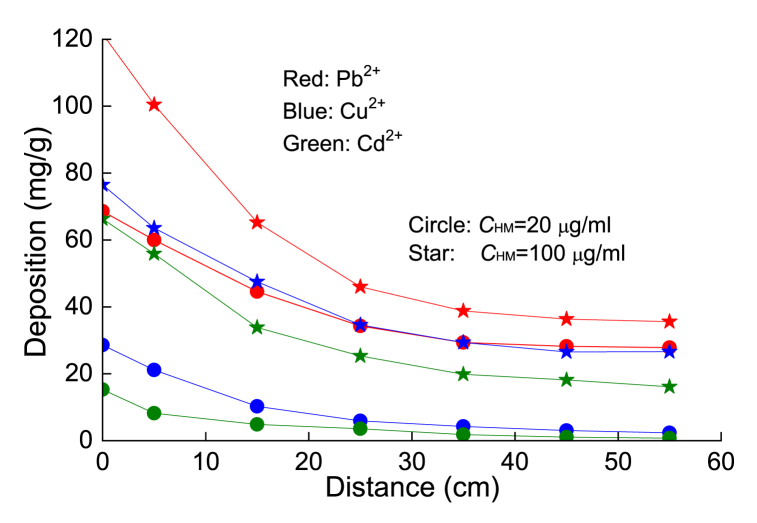
Concentration distribution of the three HMs along the injection distance at different injected concentrations.

Fig. 12 shows that the migration process of different HMs in unsaturated soil had obvious differences. Generally, the migration distance and concentration of Pb^2+^ are greater than those of Cu^2+^ and Cd^2+^, which is consistent with the strength order given by the static adsorption-desorption test. The greater the adsorption of HMs is, the greater the damage to the soil structure, resulting in obvious preferential flow in the process of seepage. Hence, the concentrations of HMs will show a significant uneven distribution in the soil column. In this way, the further away they are from the injection surface, the smaller the effluent concentration. Although the concentration distributions of Cu^2+^ and Cd^2+^ are consistent with that of Pb^2+^, their adsorption capacities are relatively small. In general, the distribution concentration of HMs in the soil column with a higher injection concentration is significantly higher than that in the case of a lower concentration.

Experimental studies on the migration of contaminants (e.g., HMs) in unsaturated soil using silty soil as porous media can provide theoretical and technical support for future remediation of strata contamination.

## 4 Conclusion

The proposed non-linear adsorption-desorption model can better fit the static adsorption-desorption experimental results, and the fitting parameters are related to the temperature, concentration and kind of HM. The equilibrium adsorption parameter increases with increasing temperature, the equilibrium desorption parameter decreases in this process, which indicating that the increase in temperature improves the adsorption performance of silty soils. The order of adsorption capacity of the three HMs is Pb^2+^ > Cu^2+^ > Cd^2+^. The microstructure of the silty soils loaded with HMs shows that the adsorption of HMs on the silty soils is closely related to the mineral compositions and functional groups in the silt microstructures. In addition to physical adsorption, the adsorption of HMs is related to the hydrolysis reaction of the mineral components kaolinite, calcite, dolomite, plagioclase and quartz. Moreover, the greater the adsorption capacity of HMs is, the larger the change in the soil surface structure, leading to a reduction in the water-holding capacity of soil particles and a decrease in the suction capacity of the matrix, which will result in preferential flow and early leaching of the filtrate. Therefore, the HM concentration first tends to increase at the initial stage and then reaches an effluent peak. When the soil is gradually saturated, the outflow concentration decreases rapidly and then tends to reach a steady state.

## Ethical approval

This article does not contain any studies with human participants or animals performed by any of the authors.

## Consent to participate

The authors agree to participate in this article.

## Consent to publish

The authors agree to publish this article.

## Data availability statement

The original data are available from the corresponding author upon reasonable request.

## CRediT authorship contribution statement

**Bing Bai:** Writing – review & editing, Writing – original draft, Methodology, Funding acquisition. **Fan Bai:** Writing – review & editing, Investigation, Conceptualization. **Jianpeng Hou:** Writing – review & editing, Investigation.

## Declaration of competing interest

The authors declare the following financial interests/personal relationships which may be considered as potential competing interests: Bing Bai reports financial support was provided by 10.13039/501100001809National Natural Science Foundation of China. If there are other authors, they declare that they have no known competing financial interests or personal relationships that could have appeared to influence the work reported in this paper.
