# Supplementary material for: The migration process and temperature effect of aqueous solutions contaminated by heavy metal ions in unsaturated silty soils
Source: Heliyon. 2024 Apr 26;10(9):e30458. doi: 10.1016/j.heliyon.2024.e30458 (PMC11077035; doi:10.1016/j.heliyon.2024.e30458)
Supplement: Multimedia component 1 [file mmc1.docx]

[Supplementary](javascript:;) data

**The migration process and temperature effect of aqueous solutions contaminated by heavy metal ions in unsaturated silty soils**

Running title: Migration process of aqueous solutions

Table. S1; Fig. S1; Fig. S2

Table S1 Parameters of the nonlinear adsorption-desorption model (i.e., Bai model)

| HMs | Temperature (°C) | *k*_d_ (ml/mg) | *β*_1_ | *R*^2^ | *k*_r_ (ml/mg) | *β*_2_ | *R*^2^ |
| --- | --- | --- | --- | --- | --- | --- | --- |
|  | 20 | 0.5694 | 0.0027 | 0.998 | 0.4735 | 0.0714 | 0.979 |
| Pb^2+^ | 40 | 0.5746 | 0.0021 | 0.993 | 0.4601 | 0.0638 | 0.990 |
|  | 60 | 0.5925 | 0.0021 | 0.993 | 0.4338 | 0.0650 | 0.969 |
|  | 20 | 0.5648 | 0.0066 | 0.995 | 0.3760 | 0.0940 | 0.945 |
| Cu^2+^ | 40 | 0.5652 | 0.0051 | 0.990 | 0.2810 | 0.0610 | 0.949 |
|  | 60 | 0.6036 | 0.0050 | 0.997 | 0.2700 | 0.0600 | 0.965 |
|  | 20 | 0.5682 | 0.0077 | 0.996 | 0.1055 | 0.0402 | 0.972 |
| Cd^2+^ | 40 | 0.5763 | 0.0051 | 0.997 | 0.0798 | 0.0328 | 0.973 |
|  | 60 | 0.6224 | 0.0060 | 0.989 | 0.0737 | 0.0248 | 0.948 |

Fig. S1 Adsorption-desorption curves of Cu^2+^: (a) 20 °C, (b) 40 °C, and (c) 60 °C

Fig. S2 Adsorption-desorption curves of Cd^2+^: (a) 20 °C, (b) 40 °C, and (c) 60 °C
